# Affordability and Non-Perfectionism in Moral Action

**DOI:** 10.1007/s10677-019-10028-4

**Published:** 2019-09-14

**Authors:** Benedict Rumbold, Victoria Charlton, Annette Rid, Polly Mitchell, James Wilson, Peter Littlejohns, Catherine Max, Albert Weale

**Affiliations:** 1 Department of Philosophy, University of Nottingham, Nottingham, UK; 2 Department of Global Health & Social Medicine, King’s College London, London, UK; 3 Department of Bioethics, The Clinical Center, U.S. National Institutes of Health, Bethesda, MD USA; 4 Department of Philosophy, University College London, London, UK; 5 School of Population Health and Environmental Sciences, King’s College London, London, UK; 6 Independent, London, UK; 7 Department of Political Science, University College London, London, UK

**Keywords:** Affordability, Total cost, Non-perfectionism, Numerical discrimination, Fairness, Lotteries, Broome

## Abstract

One rationale policy-makers sometimes give for declining to fund a service or intervention is on the grounds that it would be ‘unaffordable’, which is to say, that the total cost of providing the service or intervention for all eligible recipients would exceed the budget limit. But does the mere fact that a service or intervention is unaffordable present a reason *not* to fund it? Thus far, the philosophical literature has remained largely silent on this issue. However, in this article, we consider this kind of thinking in depth. Albeit with certain important caveats, we argue that the use of affordability criteria in matters of public financing commits what Parfit might have called a ‘mistake in moral mathematics’. First, it fails to abide by what we term a principle of ‘non-perfectionism’ in moral action: the mere fact that it is practically impossible for you to do all the good that you have reason to do does not present a reason not to do whatever good you can do. And second, when used as a means of arbitrating *between* which services to fund, affordability criteria can lead to a kind of ‘numerical discrimination’. Various attendant issues around fairness and lotteries are also discussed.

## 1

One reason public policies are sometimes rejected is on the grounds that they are ‘unaffordable’. What is meant by this varies. However, in at least one significant class of cases it is taken to imply something like the following: ‘the total cost of providing the service or intervention for all eligible recipients would exceed the budget limit’. Free school meals, for example, might be deemed ‘unaffordable’ if the total cost of providing them to every child who would be eligible would be more than the relevant budget allowed.^1^ State-funded solicitors (also known as ‘legal aid’ or ‘public defenders’) for those involved in certain civil disputes might be rejected on similar grounds.

In recent years, one place where decision-makers have begun employing this kind of argument with increasing frequency is with respect to decisions over which health care interventions to fund. Such decisions are typically made on a variety of grounds. Decision-makers (or priority-setters as they are often known in this context) might first look at whether the intervention is effective; then whether it is cost-effective; then again whether it is beneficial to those who are particularly badly off; whether there are other treatments available to those with the relevant condition; and so on.^2^ Of late, however, certain priority-setters have also begun appealing to considerations of total cost. For example, although it is widely regarded as both effective and cost-effective, some health care systems have been reticent about funding sofosbuvir, a new treatment for hepatitis C, partly on the grounds that it would have a high total cost (by virtue of the fact that it is relatively expensive and also has a high number of eligible recipients) (Kieslich et al. 2016; Gornall et al. 2016).

Is failing to fund a service or intervention on the grounds it is ‘unaffordable’ morally permissible? To date, the philosophical literature has been largely silent on this issue.^3^ However, the question of whether ‘affordability’ necessarily ought to present a bar to public funding opens up a host of interrelated normative questions, including perfectionism in moral action, matters of fairness between persons and the moral arbitrariness of group membership.

In this article we explore these matters in depth. Although our argument here has important qualifications, we conclude that, normally speaking, the use of affordability criteria is morally impermissible. First, in binary decisions as to whether or not to fund a given service, the use of such criteria fails to abide by what we call the ‘Principle of Non-Perfectionism’ in moral action, namely that the mere fact that it is practically impossible for you to do all the good that you have reason to do, does not present a reason *not* to do whatever good you can do. Second, even when employed solely as a means of deciding *between* services, we argue that the use of an affordability criterion in funding decisions ultimately leads to a kind of ‘numerical discrimination’; that is, it unjustly penalises some potential recipients for the morally arbitrary property of happening to belong to a group of a certain size.^4^

Let us begin with a few definitions. By the ‘total cost’ of a service or intervention, we understand the unit cost multiplied by the number of times it will be used within a given period (for example, a financial year), assuming it is made available to all eligible recipients within a given population. A high total cost, then, might be a function of a high unit cost and a low number of potential recipients, a low unit cost and a high number of potential recipients, or a high unit cost and a high number of potential recipients.

From here, we understand ‘affordability’ as expressing a relationship between the total cost of a service or intervention and a nominal budget. Specifically, a service or intervention may be deemed *affordable* if the total cost of funding the service or intervention falls *within* the budget limit and *unaffordable* if its total cost *exceeds* the budget limit.

These definitions suffice for our present purposes. However, it is perhaps worth noting that they also contain an ambiguity: namely, the circumstances under which the funding of a service or intervention might be viewed as exceeding the budget limit. For example, on one reading of unaffordability, a service or intervention might be considered unaffordable if its total cost is greater than the budget as a whole. By contrast, another way in which a service or intervention might be considered unaffordable is if there isn’t enough resource within the budget to fund that intervention (given its total cost) once all other financial commitments have been met.

Nothing much in our argument turns upon the difference between these two possible senses of the term ‘unaffordable’. However, the distinction is worth noting because it alerts us to the fact that appeals to affordability (and unaffordability) are often made posterior to a certain set of prior (sometimes morally-motivated) commitments. For example, ‘given we are already committed to funding/have the funding of a, b, and c, we cannot also afford d’.

## 2

What, then, might be wrong about refusing to fund a service or intervention on the grounds that it is ‘unaffordable’? Before we begin this analysis, it is helpful to make an important clarification: that is, in exploring the moral permissibility of employing affordability criteria in public finance decisions, we take the relevant *set* of decisions to be those that are, in a sense, already morally predisposed. Which is to say, we take them to be decisions with respect to which there is already at least *some* moral reason *to* fund the relevant service or intervention. What kind of reasons might these be? Our hope here is to remain as non-committal as possible, both between various potential first-order reasons and second-order moral theories. For example, we want to try to stay neutral between, say, consequentialist and non-consequentialist conceptions of what constitutes a moral reason for action. One way to put the thought here then might be to say that whatever reason *you*, dear reader, think constitutes a good reason to fund a given service or intervention, *that’s* the kind of reason we take to be both at play and demanding action in these kinds of funding decisions – whether that is because funding the relevant service would make things go best; or it would abide by a set of rules that, if accepted by everyone, would make things go best; or it would fulfil an individual’s rights; or it would fulfil an imperfect duty of beneficence or assistance on the part of the policy-maker – whatever. (It may also be worth noting here that we do not take any of the normative conclusions we reach in this article to depend on one’s subscription to any of these theories).

Given that we are already dealing with situations in which policy makers *have* some reason to fund the relevant service, then, the central question at the heart of this paper is whether *the mere fact that the service is unaffordable* constitutes a reason *not* to fund it. And here our answer is ‘no’.

Why not? The crucial point here is that affordability, as we have described it, is a function of total cost. As above, a service or intervention is ‘unaffordable’ to the extent that the total cost of providing the intervention or service for all eligible recipients would exceed the budget limit. One gloss we may put on this is to say that the use of an affordability criterion looks to whether it would be practically possible to do *all the good* (i.e. providing the intervention) that you have reason to do. And where it *is* possible to do all the good that one has reason to do, an affordability criterion sanctions funding. Where it is *not* possible, the criterion refuses funding.

It is at this point though that we get the divergence with standard moral reasoning. For typically speaking, when considering whether or not we ought to do something, we do not look to whether it is practically possible to do *all* the good that we have reason to do, but rather whether it is practically possible to do *any* good whatsoever. Moreover, where it is possible to do *some* good, rather than no good, the mere fact that it would not be possible to do all the good that we have reason to do, is not taken to constitute a reason *not* to do that little good that we can do. Consider, for example, the following, Taurek-style situation.

*Coast Guard*: Volcanic eruptions have placed the lives of many in immediate jeopardy. A large number of islanders have gathered, awaiting evacuation. A local Coast Guard evacuation ship is in the area. However, the ship only has enough seats to carry half the islanders. Should the captain head to the island anyway? (Loosely based on Taurek 1977)

In cases such as this, the answer to the question looks so obvious that it is almost not worth posing. Of course the captain should head to the island. There are islanders in peril and she is in a position to save a substantial proportion of them. Moreover, the mere fact that she wouldn’t be able to save *all* those that she has a reason to save presents no reason *not* to save those that she can.

Different moral theories are likely to offer different explanations of why our moral reasoning goes like this. For consequentialists, it may be that this is simply reflective of a deeper commitment to maximizing good outcomes. The thought here, then, is that we should not fail to do the good we *can* do, merely on the basis we cannot do all the good we have reason to do, because then we wouldn’t be maximizing the good, we wouldn’t be making things go best. For, say, patient-centred deontologists, the story may have nothing to do with maximizing anything.^5^ The point is that each of the islanders in *Coast Guard* has a *right* to be saved. And since each islander’s right engenders a binding bipolar, or directed, duty on the captain, completely independently of any other obligations the captain may be under (including those engendered by other rights-holders), the mere fact that the captain cannot fulfil her duties with respect to *all* the rights-holders on the island does not present a reason for her to fail to fulfil her duties with respect to *some* of the rights-holders.^6^ Rather, she should do for each rights-holder what she can.^7^

However, it may be that the underlying motivations here are more basic than this. That is, one way we might understand our moral preference for doing what we can is as part of a more general stance against perfectionism or idealism in moral action. The thought here then is that when we look to whether or not we can do all the good that we have reason to do, we are looking towards the viability of bringing about a perfect, or ideal state of affairs, or acting in such a way that completely or entirely fulfils our moral duty or duties. Yet there is the concern that, in doing so, we ought not to lose sight of the merely good, or bare improvement. Clearly, when we have multiple moral demands upon our action, doing everything we have reason to do would be better than only doing some of those things. However, doing something is still better than doing nothing. Moreover, the fact that doing everything *is* better than just doing something, in no way appears to imply that doing something is *not* better than doing nothing. Rather, to refuse to bring about the merely good on the grounds it fails to achieve the perfect is to fetishize ‘completeness’ in moral action, to irrationally focus our attention on ideal responses to moral problems. Common-sense morality, by contrast, looks entirely content with incremental improvements to the status quo.

We might reach, therefore, something like the following principle:

*Principle of Non-Perfectionism*: The mere fact that it is practically impossible for you to do all the good that you have reason to do, does not present a reason *not* to do whatever good you can do.

And, again, we take ‘doing good’ here – as at all points above – to be open to various competing accounts of what ‘doing good’ amounts to.

It is in respect to the Principle of Non-Perfectionism, then, that the use of an affordability criterion becomes morally impermissible. As above, by looking to matters of total cost, an affordability criterion draws us into a moral calculus entirely focused on the achievement of ‘ideal’ solutions. It effectively asks whether we could do *everything* we have reason to do for *everyone* we reason to do it for, and rules out public financing wherever that is practically impossible. In so doing it ignores the fact that – as the Principle of Non-Perfectionism attests – the mere fact that it is practically impossible for you to do all the good you have reason to do does not present a reason not to do whatever good you can do. Thus, in all those cases where it *is* possible to provide some services, or interventions, to some eligible recipients – that is, where partial provision is possible – the mere fact that a service as a whole may be *unaffordable* – it would not be possible to fund for all eligible recipients – should not be given as a reason against its funding. Rather, we should ask ourselves whether it is possible to do something for someone, even if we cannot do everything for everyone.

## 3

From the foregoing argument, then, we might conclude that refusing to fund a service or intervention on the grounds that it is ‘unaffordable’ is to make what Parfit (1984) might have called a mistake of moral mathematics: that is, it fails to recognise that the mere fact it is not possible to do everything for everyone does not, in itself, constitute a reason *not* to do something for someone. However, there is a way in which the preceding argument might be put under pressure. That is, one might claim that there are situations in which the fact it is not possible to everything for everyone *does* constitute a reason *not* to do something for someone: namely, where doing something for someone results in situations that are unacceptably *unfair*.

One way we can make sense of this idea is via Broome’s influential account of fairness (Broome 1990).^8^ For Broome, fairness requires the proportional satisfaction of claims. Equal claims require equal satisfaction and stronger claims require more satisfaction than weaker ones - though weaker claims always require some satisfaction. However, as Broome points out, one natural consequence of this view is that, where goods are indivisible and there is not enough to go around, sometimes the fairest thing to do can be to refrain from satisfying anyone’s claims whatsoever. As he puts it:

‘Now let us concentrate once more on cases where the good to be distributed is indivisible, and there is not enough to go round. Take a case, first, where all candidates have equal claims. It would be possible to satisfy their claims equally, as fairness requires, by denying the good to all of them. There may be occasions when it is so important to be fair that this is the right thing to do.’

Considerations of fairness, therefore, might justify doing less than we would otherwise have reason to.

One way we might summarise Broome’s view here is by way of what Keynes - reported via Bernard Williams – apparently used to call the Principle of Equal Unfairness:

*Principle of Equal Unfairness*: if you can't do a good turn to everybody in a certain situation, you shouldn't do it to anybody. (Keynes as quoted by Williams in Williams 1973, 226)

Both Keynes’s title for the principle and the context within it was raised – Williams reports that Keynes was prone to using it in relation to deliberations of academic bodies – suggests that he did not consider it a particularly appealing maxim.^9^ Williams is similarly unimpressed, writing:

There are indeed human activities and relations in which impartiality and consistency are very much the point. But to raise on these notions a model of all moral relations is, just as Kant said it was, to make us each into a Supreme Legislator; a fantasy which represents, not the moral ideal, but the deification of man. (Williams 1973, 226)

We might also think that, insofar as we take the ‘good turns’ referred to in the principle as the fulfilment of rights-generated duties, any patient-centred deontologists are unlikely to find the principle attractive. For such thinkers (or at least, many of them), there is no virtue in any situation in which rights go unfulfilled, regardless of whether or not non-fulfilment would maintain some relation of ‘fairness’ between rights-holders, or achieve some value of equality, or whatever else. Rather, in line with the reasoning laid out in the previous section, rights demand action on the part of duty-bearers in pair-wise connections, the existence of multiple such connections in no way weakening the demands of any one of them, nor the fulfilment of any duties on the part of the duty-bearer doing any ‘wrong’ to any rights-holder who finds their duty as yet unfulfilled (at least, beyond the bare fact the duties engendered by their rights remain unfulfilled).

However, along with Broome, we are reasonably sympathetic to the idea that the Principle of Equal Unfairness might have some truth to it; that, in some situations at least, there is a value in maintaining fairness between parties (or at least, not causing *greater* unfairness); and that, in principle, there may be situations in which considerations of fairness could be such that, all things considered, we ought to do less than we could. In short, the fact that a service could only ever be partially provided could justify our failing to provide any of that service to anyone whatsoever. In turn, this would suggest that, on certain occasions, policy-makers may be all-things-considered justified in denying public funding on the grounds that the service or intervention is ‘unaffordable’. i.e. that the total cost of funding the service or intervention for *all* eligible recipients would exceed the budget limit.

However, before one imagines that this flatly contradicts the conclusions of the previous section there are a number of points worth making. First, opening ourselves to the possibility that considerations of fairness *could* sometimes justify our doing less than we have reason to in no way falsifies the idea that, generally speaking, we ought to do something rather than nothing, or the Principle of Non-Perfectionism. Rather, at most, it only shows that in certain circumstances reasons of fairness can be such that the right thing to do, *all things considered*, is less than we (otherwise) have reason to, and that this is true in spite of the reasons we have to do what we can.

Second, it is also worth noting that even the Principle of Equal Unfairness is not a direct opponent of the Principle of Non-Perfectionism. Most notably, the Principle of Non-Perfectionism is concerned with any case wherein one attempts to justify failing to do whatever good one can do merely on the basis it is practically impossible for you to do all the good you have reason to. Such cases may well include cases in which it is practically impossible for you to do all the good you have reason to do because you cannot do something for everyone. However, they are also likely to cover other sorts of cases. That is, it might be that you cannot do all the good you have reason to do *not* because there is something you cannot do for everyone, but because you cannot do everything for someone. (Here we might consider, for example, cases in which one has multiple duties with respect to a single individual yet only the resources to fulfil some of them). Those who endorse the Principle of Equal Unfairness, then, are likely to disagree with the Principle of Non-Perfection insofar as it rules out failing to do something for anyone on the grounds it would be practically impossible to do that same thing for everyone. However, they might at the same time willingly endorse the Principle of Non-Perfectionism when it comes to ruling out failing to do something for someone simply by virtue of it being practically impossible to do everything for that person. As above, the underlying motivation behind the Principle of Equal Unfairness (as we read it at least) is the maintenance of fairness between persons. Yet *that* motivation would provide no grounds for ruling out failing to do something for a single individual, simply on the grounds one could not do everything for that individual. To put it in Broome’s terms: the Principle of Equal Unfairness applies when partial provision results in the unequal satisfaction of claims *across persons*. It does not seem thereby to have much to say at all about the unequal satisfaction of claims with respect to a single person.

Third, although it does seem possible that, in certain circumstances, for reasons of fairness, the right thing to do might be to refuse to do ‘a good turn’ to anyone whatsoever, it is far less clear how frequent such situations are. In *Coast Guard*, for example, we appear to be presented with a clear case in which the precisely the opposite reasoning applies. That is, we would normally think it would be appalling if the captain were to refuse to save any islanders merely on the basis that she could only do something for some of them, rather than something for all of them (her ship only being able to carry half the islanders). Rather, normally speaking, we would think that the captain *should* save whatever islanders she can, regardless of whether it entails some ‘unfairness’ in Broome’s sense (hence the possibility, intimated earlier, that there may be outcomes that are *acceptably unfair*). Indeed, Broome’s own view appears to be that *this* latter situation is the far more common, writing:

[any failure to do what one can] would totally fail to meet the satisfaction requirement, and normally the demands of fairness will not be enough to outweigh this requirement completely. It will be better to use as much of the good as is available. (Broome 1990, 97).

Of course, what we really need here is a thorough investigation of *when* resulting unfairness *does* present a reason not to satisfy claims and why it does when it does.^10^ Unfortunately, such an investigation takes us beyond our main concerns in this paper. Rather, for now we might simply conclude that although considerations of fairness *do* look like one possible reason why an unaffordability criterion might, in fact, be morally permissible, in order for policy-makers to *defend* the use of such a criterion on the grounds of fairness, several conditions would need to be met. First, that the partial provision of the relevant service or intervention would be likely to result in unfairness *across people*, rather than the less-concerning-to-fairness possibility of unequal satisfaction of claims with respect to a single person. Second, that facts of the case at hand are sufficient to warrant the conclusion that it would be better, all things considered, to preserve whatever fairness exists by doing less than one could, than generate any new unfairness by disproportionately doing a ‘good turn’ to some, rather than others. Finally, depending on whether one subscribes to the patient-centred deontologists’ point about rights, that the ‘good turn’ we are doing is not the fulfilment of an individual’s *rights* (where considerations of fairness ought to prove no bar to partial provision) but are, instead, premised on some other moral reason – say the maximization of the good, or a unipolar, or undirected duty of beneficence.

With all this in mind, then, the argument-from-fairness does place a significant caveat on our position. It is not true that the mere fact that a service or intervention is ‘unaffordable’ does not constitute a reason not to fund it, for there are clearly cases in which the fact that a service or intervention is ‘unaffordable’ *does* constitute a reason not to fund it (i.e. those where the demands of fairness speak decisively against partial provision). At the same time, however, it also appears clear that, such is the strictness of the conditions under which this kind of argument could be made, it is *generally true* that policy-makers ought not deny funding to an intervention merely because its total cost is such that it is practically impossible for us to provide it for all those who would be eligible. Rather, in line with the Principle of Non-Perfection, in a significant number of cases, we should still do what we can.

## 4

Before we continue, there is another point worth acknowledging here – one that provides a further argumentative hurdle to those who might otherwise want to defend the use of an affordability criterion on grounds of fairness. For contrary to the line of argument gestured to at the end of the preceding section, it is not entirely clear that partial provision always *does* undermine fairness – or undermine it completely. Rather, provided we abide by certain procedural mechanisms in the satisfaction of whatever claims we can, we might think a provision can still be fair even when it is partial – which is to say, even when it results in some people not getting what they ought, while others do. One such mechanism, for example, might be the distribution of goods via a lottery.

Needless to say, the debate about what, precisely, makes lotteries fair continues (see, e.g., Broome 1984, 1990; Stone 2007; Saunders 2008, 2009). However, for one defence we might return to Broome’s account of fairness. (Indeed, Broome himself considers the ability of his account to justify the fairness of lotteries as among his account’s chief virtues (Broome 1990)). On Broome’s view, where we use a lottery to decide who gets what, we always create a certain degree of unfairness simply by virtue of the fact that, despite all having equal claims, the claims of some candidates are satisfied where others are not. However, at the same time, Broome argues that, insofar as each is given an equal *chance* of getting the good, then each candidate still receives a sort of ‘surrogate’ satisfaction of their claim. In this way, then, Broome concludes that although lotteries do not entail perfect fairness, they do at least ensure that the demands of fairness are met to some extent (Broome 1990, 97-8).^11^

If we assume, therefore, that it is possible for a provision to be both partial and fair – say, when who gets what is decided by lottery – then it is not true that considerations of fairness always rule out partial provision. Rather, properly speaking we ought to say that partial provision – and hence unaffordable services or interventions – should only be ruled out on the grounds of fairness if it can be shown that: i) the partial provision of the relevant service or intervention would be likely to result in unfairness *across people;* ii) considerations of fairness are such that *in this case,* all things considered, we ought to do less than we could; iii) (potentially) the relevant ‘good turn’ is not a matter of the fulfilment of individuals’ *rights* (as per the conclusion of section 3); *and* that iv) it is not possible to achieve a kind of ‘surrogate’ fairness by other means (e.g. distribution-through-lottery). In other words, if we accept that distribution by lottery *can* be fair, we might similarly say that the use of an affordability criterion can only be justified when holding a lottery to see who gets what would *not* be possible.

## 5

Based on the arguments presented in 3, 4 and 5, therefore, we might conclude that in at least a significant proportion of cases, it is unacceptable to refrain from the provision of a service merely on the grounds that it is unaffordable – that its total cost would exceed the budget limit. Rather, we ought to do what we can. However, if this suggests that, generally speaking, we shouldn’t use affordability as a basis for deciding whether we ought to provide a service or not, what about those cases where we are choosing *between* which services to fund?

One reason this question is important is that it more closely mirrors many of the dilemmas decision-makers actually face. That is, often decision-makers are not tasked with deciding whether or not to fund service x, given a certain fixed budget, (or what remains of their budget after other commitments have been met), but rather which out of a range of possible services or interventions they ought to fund with the resources they have available. Might one argue, then, that while affordability shouldn’t be used in binary decisions about whether a service should be funded or not, it can be used when deciding between multiple funding options?

One thing that is perhaps worth noting here is that such a suggestion does not fall afoul of the same arguments we made against the use of affordability criteria in the binary case. After all, it is not as if, when using an affordability criterion to decide *between* which services we ought to fund, we are ever *failing* to do what we have reason to do (as we would be, were we to refuse to do something for someone merely on the basis we could not do everything for everyone). Rather, it is simply that we are deciding *which* of our many moral duties, to many different individuals, to fulfil, and using affordability as a criterion for making that decision. But, whatever we eventually decide, we are doing precisely the same amount of good.

Indeed, far from looking obviously wrong, we might even think that there is something attractive about a decision-rule that prioritises the ‘affordable’ choice. That is, when we fund the affordable option, we at least ensure that all eligible recipients get what they need. By contrast, when we fund the unaffordable option, we are always left with a rump of disgruntled candidates, those that, despite having been eligible, have not got what they need (whether or not they have also had the ‘surrogate satisfaction’ of a lottery).

Put like this, it is difficult not to feel a natural preponderance for the former kind of outcome over the latter. One thought here, picked up by Unger, is that we often seem to prefer a ‘clean scene’ to a ‘continuing mess’:

Related to thoughts of the multitude, there's the thought of the continuing mess: “Even if I do send the $100 to UNICEF, there'll still be many children very prematurely dying…. [By contrast] the Sedan's trespasser [in urgent need of medical help] presented me with a particular distinct problem. If only I got him to the hospital, the problem would have been completely resolved. Starting with just such a problem, I'd finish with nothing less than a completely cleaned scene. How very different that is from the continuing mess involving all the distant children!” (Unger 1996, 41).

However, as Unger goes on to point out, there is an obvious error in this kind of reasoning. In dealing with the individual, we no more ‘clean the scene’ than dealing with multitude. In reality, every individual in peril is part of the ‘continuing mess of the world’. In a similar way, in opting for the ‘affordable’ option, we no more ‘clean the scene’ than if we opt for the unaffordable one, for in both cases we are still left with a group of recipients that have not got what they need. Given that, by definition, our dilemma involves a situation where we are faced with multiple legitimate claims upon our action and cannot meet them all, no matter what we do we will always be left with a continuing mess in one direction or another.

In reality, far from being a morally attractive option – or even a morally neutral one - using an affordability criterion as a means to determine between services looks morally impermissible insofar as it appears to unjustly penalise some potential recipients for the morally arbitrary property of happening to belong to a group of a certain size. To see this, consider:

*Coast Guard 2*: Volcanic eruptions have placed the lives of many in immediate jeopardy. A large number of islanders are gathered at the north end of the island, awaiting evacuation. A handful find themselves on the southern tip. The captain of the only Coast Guard evacuation ship in the area finds herself midway between the two. Her ship has enough seats to carry all the islanders on the southern tip but only half of the islanders on the northern tip. Where should she head first? (Loosely based on Taurek 1977)

Now, as these kinds of examples are used in the literature around aggregation, the central question is whether the captain has a moral obligation, where possible, to save the greater number and, if not, how she ought to decide between the two groups. However, for our present purposes, the question of whether the captain ought to save the greater number is largely irrelevant. This is because, where matters of affordability are concerned, there is no question about saving the greater number. Indeed, if we are basing our decision over which group to save on which we can *afford* – read: which group it would be possible to save *in its entirety* – the fact that there are *more* northerners on the northern tip constitutes a reason *not* to sail there, since it would not be possible – read: unaffordable – to save all the islanders there assembled.

For our present purposes, then, the real question is: *does the mere fact that the Coast Guard’s ship cannot carry all the islanders on the northern tip give her reason to head to the southern tip*? And here it seems quite plain that it does not. That is, all else being equal, we have no greater reason to save P, an islander on the southern tip, than Q, an islander on the northern tip, simply by virtue of the fact that i) P happens to belong to a smaller group of people, and ii) we wouldn’t be able to save all the people on the northern tip. Rather, in such a situation, Q might justifiably say to any captain minded to head south on the basis of i) and ii): ‘Well, even if you can’t save all of us, why not just save half? Why should the fact that you can *only* save half make any of us any less deserving of rescue than any of the islanders in the south?’^12^

What these examples highlight, of course, is that where we can fund services and interventions for some and not all, the fact that it is sometimes possible for us to fund such services for all of one group rather than some of another is not a sufficient reason to discriminate between them. Rather, what matters in these kinds of cases is simply our duty to each individual by virtue of their present situation. Thus, where we find that all the relevant individuals are, morally speaking, in the *same* situation, as in the case of *Coast Guard 2*, the mere fact that they happen to be split into two groups – northerners and southerners – changes nothing about our duties towards them. Certainly, we do not fulfil our moral duties any more or less by saving *all* of one arbitrary grouping as opposed to *half* of another. If we save five southerners or five northerners, the moral situation is the same – we have fulfilled our duties to precisely the same extent – whether or not that means we have saved ‘all’ the southerners or only ‘half’ the northerners.^13^

The fact, then, that the captain would be able to save *all* the southerners (and only some of the northerners) is not, in itself, a reason to save the southerners over the northerners. And if *this* is right, then we cannot prioritise funding ‘affordable’ services and interventions over ‘unaffordable’ ones solely on the grounds that we can offer full provision in the first case but only partial provision in the second.

We might, then, reach something like the following principle:

*The Impermissibility of Numerical Discrimination (IND)*: All other things being equal, it is not morally permissible to prioritise between groups merely on the basis that we might meet the needs of *all* those in one group as opposed to *some* in another.

## 6

Before continuing, it is perhaps worth re-iterating that these conclusions are not simply of interest at the level of value theory. Rather, as intimated in the introduction to the piece, since policy-makers are often prone to appeal to matters of affordability in public financing decisions, they have a certain amount of practical significance.

For example, consider the case of infliximab (Charlton et al. 2017). Infliximab is a drug licensed for acute ulcerative colitis and severe active Crohn’s disease—the two main forms of inflammatory bowel disease, whose symptoms are very similar. When assessed by the National Institute for Health and Care Excellence (NICE) in the UK, infliximab was found to be cost-effective for both indications. However, the numbers of patients affected by acute ulcerative colitis and severe active Crohn’s disease differ substantially, with far more patients in the latter diagnostic group.

Let us suppose that some set of decision-makers were forced to choose between funding infliximab for either acute ulcerative colitis or severe active Crohn’s disease. The question, then, is whether, in making such a decision, they may appeal to considerations of affordability. And the answer from this analysis is ‘no’. That is, following the logic above, funding infliximab for acute ulcerative colitis over funding it for severe active Crohn’s disease simply on the grounds that, say, the former is ‘affordable’ and the latter not, clearly presents a case of ‘numerical discrimination’. All other things being equal, the mere fact that P has condition Z – acute ulcerative colitis – that happens to affect only a few people, does not give us any greater reason to save them than to save Q, someone who has a condition Z* – severe active Crohn’s disease – that happens to affect a lot of people. Again, as in *Coast Guard 2*, Q might very well ask the decision-maker, ‘Well, if you cannot afford, by the fixed terms of your budget, to benefit all the people with severe active Crohn’s disease, why not just benefit those of us you can afford to? Why should the fact that you can *only* benefit half make any of us any less deserving of funding than those with acute ulcerative colitis?’

## 7

Let us take stock for a moment. Thus far we have argued that, in matters of public policy, the fact that a given service or intervention may be unaffordable – which is to say, its total cost, if provided to all eligible recipients, would exceed the budget limit – does not present a reason not to fund it. Rather, when it is possible to provide the service to at least some, we ought to do what we can (Section 2). At the same time, however, we have recognised at least one set of cases where this dictum does not apply: namely, those in which, for reasons of fairness, the right thing to do is less than we could. These cases being admitted, though, we have gone on to argue that anyone attempting to defend the use of affordability criteria on the grounds of fairness have a number of hurdles to overcome (conditions i) to iv), Section 3 and 4). Most recently, we have argued that as well as morality (normally) counselling against the use of an affordability criterion in binary decisions about whether or not to fund a given service or intervention, it also counsels against using such a criterion in deciding between which services we ought to fund. Rather, as we have shown, making decisions between services on the grounds of affordability violates IND (Section 5).

This last point, however, leads to a natural question: if one of the conclusions of the preceding analysis is that it is morally impermissible to use affordability as a means of discriminating between two funding options, what ought we to do when we are presented with two different funding options, A and B, which are identical in all morally relevant respects and yet one is affordable and another is not?

Of course, a point one might raise here is whether it is ever really likely that two different funding options would be alike in all morally relevant respects, so that the only difference between them is that one is affordable and the other is not. After all, if we go back to the case of infliximab cited earlier, we might note that there are highly likely to be differences between the two groups which might, on their own, persuade decision-makers to prioritise one group over the other beyond difference in affordability.

However, assuming that two candidate funding options – A and B – really *are* alike in all relevant respects other than the fact that funding A would be affordable and funding B would not, how ought decision-makers approach that decision? In our view, there is at least one morally permissible response available: namely, for decision-makers to group all the potential recipients under A and B together and hold a lottery to see who gets what.^14^

Our reasoning here builds on justifications of distribution-by-lottery cited earlier. That is, where we know, as stipulated, that each person’s claim to resources under A or B is precisely equal to everyone else’s, the fairest thing to do would appear to be to give *all* the potential recipients under both A and B the same claim to the relevant resource and leave the actual allocation across the population to chance.

This suggestion might be challenged. For example, it might be argued that, against our suggestion to the contrary, such a system fails to treat patients fairly. That is, it is generally accepted that one of the founding principles of justice is what is sometimes referred to as a ‘formal equality principle’: namely that we should ‘treat like cases as like’ (such a principle can be dated back to Aristotle – see Aristotle 1984a, V.3. 1131a10-b15; 1984b, III.9.1280 a8-15, III. 12. 1282b18-23). Under the foregoing decision procedure, though, it is highly likely that two individuals, each with differing needs, may be treated differently. For example, if a lottery-for-infliximab were held between patients with acute ulcerative colitis and those with severe active Crohn’s disease, one patient with acute ulcerative colitis may be given infliximab where another would not. At the same time, patients who *are* different may be treated the same. One patient with severe active Crohn’s disease may be given the same chance of treatment as one with acute ulcerative colitis. Since, however, ‘like cases ought to be treated as like’, and unlike cases ought to be treated as unlike, such a procedure violates basic tenets of fairness.

In response to this, it should, of course, be admitted that were, say, a lottery-for-treatment to be held across multiple diagnostic groups, it is indeed highly likely that two individuals, both experiencing the same condition, may not receive the same treatment, and some experiencing different conditions, may be treated the same. However, contrary to the counter above, we would contest the idea that such a situation presents an injustice.

In any evaluation as to whether a given procedure violates the formal equality principle the fundamental questions, of course, are: i) in what sense are the relevant cases considered ‘alike’; and ii) is that likeness morally relevant to the distribution at hand? What the formal equality principle demands of us with respect to distributive justice, then, is *not* that we treat any cases sharing any property in common alike (and any cases exhibiting different properties differently) but rather that we treat as like all cases which are*, in the morally relevant respects,* alike (and cases *exhibiting morally relevant differences*, differently).

The argument, therefore, that the above model *violates* the formal equality principle necessarily rests on the claim that an individuals’ membership of a certain group – e.g. a diagnostic group – is, in itself, a morally relevant property to the distribution at hand (such that the treatment of members differently to others is unfair, as is the treatment of non-members on the same terms as members). However, it is hard to see how such an argument might go. Of course, with respect to diagnostic groups, it is true that generally speaking membership of different diagnostic groups might be a good indication that the individuals within those groups are in a different kind of situation, morally speaking, from those outside such groups (a situation further complicated by the fact that one’s membership of a certain diagnostic group can sometimes *cause* one to be treated differently from non-members). However, the mere fact that one’s membership of a particular diagnostic group can sometimes indicate something about one’s moral situation does not mean that their membership *of* that group is morally relevant *in and of itself*. Where we have already stipulated, therefore, that two groups, A and B, are morally alike in all relevant respects, there is no unfairness in treating members in both groups on the same terms.

## 8

We believe the preceding analysis gets the moral theory around affordability right: namely, that the mere fact a public service is unaffordable does not necessarily present a reason not to fund it. However, at the same time, there are various considerations that may seem to counter this view.

Most notably, perhaps, much of the previous analysis has rested on the assumption that, in those situations where it is not possible to meet the total cost of a given service or intervention for all eligible recipients, decision-makers might still have the option of providing that service or intervention for *some* eligible recipients. From here, it was then surmised that unaffordability should not prove a bar to *some* degree of funding; rather, doing something is better than doing nothing.

There are two situations, however, where this logic does not hold. First, it may be the case that there are some services which are unaffordable both in that their total cost, if provided to *all* potential recipients, would exceed the budget limit but also in that their total cost, if provided to *any* potential recipients, would exceed the budget limit. For example, in cases where a service or intervention has a high total cost not because it has low unit cost and a high number of potential recipients (perhaps the default assumption of this paper), but because it has a high unit cost and a low number of potential recipients, providing the relevant service to even one recipient may be beyond the bounds of the budget limit.^15^

Another complication to be dealt with here are situations in which the benefits garnered by a given service or intervention only accrue when it is provided to a whole population. Consider, for example, a vaccination programme. One way vaccinations can be of benefit to a population is by securing herd immunity. Provided a high enough percentage of the population is protected through vaccination, the spread of a disease may be reduced because there are too few susceptible people left to infect. Given the way vaccinations work, therefore, although it may be both affordable and possible to provide a service or intervention to a certain proportion of the population, and it may benefit those who receive it, there may be little point in doing so – or at least, the resources may be better spent elsewhere. Thus, by extension, the fact that it would be unaffordable to provide a vaccination programme for all eligible recipients may be a good basis upon which to reject the whole programme, even if some level of partial provision may be both possible and of benefit.

Both these situations present cases in which a service’s unaffordability may present a bar to its funding. However, we do not believe they necessarily offer a problematic counter to the present argument. The primary aim of the present argument has been to show that, in public policy decisions, we ought not pay too close attention to whether we can provide a service to all of the people, all of the time. Rather, recognising the possibility of partial provision, we ought instead to do what we can. Thus, the fact that it is sometimes not possible to fund even a partial provision – as in the high-unit-cost/low-number-of-recipients case – does not suggest a flaw in this reasoning, for the reasoning was always directed towards those situations where partial provision was possible. Similarly, the fact that it is sometimes senseless to fund a partial provision – as in the vaccination case – also does not suggest this reasoning was mistaken, only that it is directed to those cases where partial provision is both possible and makes sense. To put it another way, therefore, such counters do not necessarily refute the present argument so much as identify its necessary conditions.

## 9

One last point before we conclude. In arguing that affordability ought not necessarily present a bar to funding certain services and interventions we do not also thereby mean that decision-makers should never consider matters of total cost.

One reason, for example, why it might make sense for decision-makers to know the total cost of a service or intervention is in respect to questions of displacement. Imagine, for example, that all resources within a health care system’s budget have already been spoken for – there is no money left to fund new entrants. Still, if a potential new entrant is deemed to be sufficiently ‘high priority’, decision-makers might decide to *make* finance available by decommissioning presently-funded, low-priority treatments and services. In such cases, then, it becomes necessary to know the total cost of an intervention in order to know how many other, low-priority interventions need to be decommissioned to free up the necessary funding. Given x’s total costs, which services would we need to displace?

To a certain extent, this is analogous to how NICE has sought to use a total cost criterion with respect to its decisions about which treatments to include within the English National Health Service. Thus, under new rules introduced in April 2017, NICE now applies a ‘budget impact test’ according to which there may be a delay in introduction of any new technology of up to three years where it is found to have an annual budget impact exceeding £20 million (NICE 2017). One way one might defend the imposition of this rule, then, is that by instituting a delay for interventions over a certain total cost, the health service is given time to identify candidates for displacement and at least begin the process of decommissioning them. (Although we ought to note that it is not entirely clear that this was NICE’s reasoning, and that there are other reasons to think that NICE’s rule is problematic – see, e.g. Charlton et al. 2017).

## 10

There is a view, perhaps, that once a service or intervention has been deemed unaffordable, that fact is sufficient to show that it shouldn’t be funded. In this article we have challenged this kind of thinking. We have argued that failing to fund a service merely on the grounds of affordability makes two errors in moral mathematics. First, in binary choices where the question is whether to fund the intervention or not, the use of such a criterion fails to recognise that, even if you cannot do everything for everyone, doing something is better than doing nothing. Second, in more complex cases where the question is whether to fund one intervention rather than another, the use of such a criterion has the potential to lead to a kind of ‘numerical discrimination’, whereby certain individuals are unjustly penalised on the morally arbitrary grounds that they happen to belong to a group of a certain size. Subject to certain important caveats around issues of fairness and the possibility of distribution-by-lottery, we have claimed that these arguments show that, generally speaking, the mere fact that a service or intervention is ‘unaffordable’ does not constitute a reason not to fund it.

## Footnotes

1 Note: in saying that one way free school meals might be deemed ‘unaffordable’ is if the total cost of providing them to every child was more than the relevant budget allowed, we mean every *eligible* child, rather than every child simpliciter. The thought here, then, is that all public policy involving the provision or supply of some service or intervention to a respective population will include some set of eligibility criteria detailing who, precisely, will be eligible for that service. Thus, in the UK at present, all children in government-funded education receive free school meals until Year Two (first grade in many other countries). After this point, children are entitled to free school meals if their parents meet a further set of criteria: e.g. they receive Income Support; income-based Jobseeker’s Allowance; support under Part VI of the Immigration and Asylum Act 1999; and so on and so forth. These are the kind of rules, then, that we understand as eligibility criteria. Moreover, in referring to the idea that a service or provision might be unaffordable if its provision was more than the relevant budget allowed, we mean that it would be unaffordable were it provided to all those that *would be* eligible for it, given whatever eligibility criteria are taken to apply in the case in hand.

2 For an analysis of one contemporary priority-setting process see, e.g., Rumbold et al. 2017.

3 To a certain extent, one might think that this issue relates to questions of *feasibility*: does, for example, the fact that the funding of a service or intervention would be ‘unfeasible’ suggest it ought not to be funded? However, as we understand it, the issues around affordability are somewhat different to those that have traditionally been discussed in the philosophical literature on feasibility. That is, traditionally speaking, at least, questions of feasibility are often concerned with issues of demandingness. Here we might think, for example, of a Rawlsian understanding of feasibility as relating to ensuring that the principles of justice do not ask ‘too much’ of a given society (understood in various ways). Questions about affordability, however, are different. Here the issue is not whether morality asks ‘too much’ of us, given our resources, but how we ought to respond to legitimate moral reasons for action. More specifically, the question is whether the sheer number of demands upon our action can, in itself, present us with a reason not to act in any way whatsoever. In this sense, questions about the ethics of affordability are perhaps more closely related to discussions about fairness. See here, for example, Williams’s reference to ‘the Principle of Equal Unfairness’ – i.e. if you can't do a good turn to everybody in a certain situation, you shouldn't do it to anybody (Williams 1973, 226). Further elaboration as to how our current subject fits with this literature can be found in Section 3.

4 The argument that affordability presents a kind of ‘numerical discrimination’ is one that we have previously made elsewhere (see Charlton et al. 2017). Unfortunately, due to limitations of space in that article, we were not able to spell our argument out there in as much detail as we would like. One aim of this paper, then, is to provide a more extensive philosophical defence of that position, as well as to alert readers to the fact that the issue is a more general one than that paper suggested.

5 For the avoidance of confusion, by ‘patient’ here we mean something like ‘recipient’ rather than anything relating to health care. In this, then, we follow a standard distinction in moral theory between ‘patient-centred’ and ‘agent-centred’ deonotologies (see, e.g., Alexander and Moore 2016).

6 For description of such duties as ‘bipolar’ see e.g., Darwall 2013, 20-2. For description of them as ‘directed’ see, e.g., Cruft 2013. Also, see these references for contrast with ‘unipolar’ or ‘undirected’ duties.

7 Not to belabour the point, but as is hopefully clear from above, we take this kind of reasoning to be entirely distinct from anything that might approximate a ‘utilitarianism of rights’ (cf. Nozick 1974). The point here is not that the captain has a duty to maximize fulfilment of rights, or minimize their non-fulfilment, but merely that each islander has a right to be saved and that right demands action on the part of the captain, independently of any other obligations that the captain may, or may not, be under.

8 Here we note Broome’s conception of fairness as influential but this should not be read as synonymous with uncontroversial. For criticisms see, for example, Hooker 2005.

9 Another writer, for example, might have simply called it the Principle of Maintaining Fairness.

10 Broome’s own response to this, as quoted above, is that what is doing the work in these kinds of cases is the relative importance of securing a fair outcome. As he puts it, ‘[t]here may be occasions when it is so important to be fair that [doing less than you can] is the right thing to do’ (Broome 1990). However, there are reasons to think that this doesn’t get things quite right. For more on this and for an alternative suggestion see: Rumbold Forthcoming.

11 In a recent helpful article, Christian Piller explains Broome’s view as follows: ‘Distributing goods by lotteries offers a fairness gain when compared to one party simply getting the good: there is a sense in which we treat equal claims equally when holding a lottery. Thus, lotteries are strictly preferable to the alternative of simply giving the good to one of the parties. Distributing goods by lotteries results in a fairness loss when compared to withholding the good: ensuring an unequal distribution means we have not treated equal claims equally. Whether we should use a lottery depends on whether the benefit of the good outweighs this fairness loss. In Broome’s view, there is no algorithm that could relieve us of having to judge each situation in this regard on its own merits.’ (Piller 2017)

12 There is, perhaps, one way the captain might defend her decision to head south in response to Q’s question. That is, the captain might argue that heading north requires her to make two arbitrary decisions: first, whether to save northerners or southerners; and second, which northerners to save. By contrast, heading south requires her to make only one arbitrary decision: whether to save northerners or southerners. On the grounds, then, that, all-else-being-equal, it is better to make one arbitrary decision than two, the captain might justifiably choose to save all the southerners rather than some of the northerners. This suggestion, put to us by one of the anonymous reviewers of the piece, seems a strong counter to the line of argument suggested above. However, we are not sure that it entirely succeeds. In particular, it is not clear why the captain needs to engage in the two-step decision process outlined above. Rather, the captain may simply decide to save the first x number of islanders she can and then flip a coin as to whether to head north or south. In doing so, it may also be that the first x number of islanders she saves are all the islanders on the southern tip of the island. However, in heading south it is not clear that she would have thereby made any fewer arbitrary decisions than were she to have headed north. It might also be argued that, even if the captain were to employ the kind of double-step decision procedure outlined above, then under the formal equality principle, she would be obliged to apply any decision rule she elected in order to decide between northerners (i.e. at point (ii)), to her decision between northerners and southerners. Otherwise any northerner penalised by that rule might legitimately complain that her case was not being treated as ‘like’ other morally identical cases. (If the decision rule, for example, was to take every third northerner until the ship was full, then why should that apply only to the northerners? Why should the decision rule not be to take every third *islander* until the ship was full?). For more on this kind of thought, and the formal equality principle, see Section 7 below.

13 We make this argument in respect to our ‘duties’ to the islanders. However, we take it that a similar point applies for those who prefer to talk about realising certain states of affairs. Indeed, on a consequentialist ethics the point seems even clearer: there is no difference between saving some of one group and all of another where the numbers are equal. Why? Because in both situations you do precisely the same amount of good.

14 Here we appreciate that the actual appropriateness and fairness of distribution-by-lottery may ultimately depend on the context and the way they are constructed. Take free school meals, for instance. Using a lottery to determine who gets free school meals seems manifestly unfair if the lottery occurs at the beginning of a child’s school career, and determines whether they get free meals for the duration of their time in school. Using a lottery to determine who gets free school meals might be less unfair if a new lottery occurs every day. The latter means that every child will get at least some free school meals, even if no child is likely to get them all the time. The situation is different with certain kinds of medical treatment. It wouldn’t make sense to have a lottery every day to see who gets to take their antibiotics, as taking a full course of antibiotics is essential for eliminating an infection and reducing the risk of antimicrobial resistance. But it might make sense with some other drugs which provide a reduced benefit, but still a benefit, when patients take a less-than-optimal dose (e.g. analgesics for mild pain). The fairness of a lottery might also depend on the remaining options for those who are not selected.

15 As an aside, it is perhaps worth noting here that costs per unit price may shift as the number of recipients change. Since there are often economies that can be made when a service or intervention is provided at scale, it may be that the unit price for a single individual is affordable when the assumption is that the same service will be provided to multiple recipients and yet is not affordable when it is only provided to a single recipient. To be clear, this fact does not threaten the arguments laid out above; however, it does suggest a reason why partial provision may not always be more affordable than comprehensive provision.
